# Valence, arousal, familiarity, concreteness, and imageability ratings for 292 two-character Chinese nouns in Cantonese speakers in Hong Kong

**DOI:** 10.1371/journal.pone.0174569

**Published:** 2017-03-27

**Authors:** Lydia T. S. Yee

**Affiliations:** 1 Department of Psychology, The Education University of Hong Kong, Tai Po, Hong Kong; 2 Centre for Psychosocial Health, The Education University of Hong Kong, Tai Po, Hong Kong; 3 Centre for Brain and Education, The Education University of Hong Kong, Tai Po, Hong Kong; Boston Children's Hospital / Harvard Medical School, UNITED STATES

## Abstract

Words are frequently used as stimuli in cognitive psychology experiments, for example, in recognition memory studies. In these experiments, it is often desirable to control for the words’ psycholinguistic properties because differences in such properties across experimental conditions might introduce undesirable confounds. In order to avoid confounds, studies typically check to see if various affective and lexico-semantic properties are matched across experimental conditions, and so databases that contain values for these properties are needed. While word ratings for these variables exist in English and other European languages, ratings for Chinese words are not comprehensive. In particular, while ratings for single characters exist, ratings for two-character words—which often have different meanings than their constituent characters, are scarce. In this study, ratings for 292 two-character Chinese nouns were obtained from Cantonese speakers in Hong Kong. Affective variables, including valence and arousal, and lexico-semantic variables, including familiarity, concreteness, and imageability, were rated in the study. The words were selected from a film subtitle database containing word frequency information that could be extracted and listed alongside the resulting ratings. Overall, the subjective ratings showed good reliability across all rated dimensions, as well as good reliability within and between the different groups of participants who each rated a subset of the words. Moreover, several well-established relationships between the variables found consistently in other languages were also observed in this study, demonstrating that the ratings are valid. The resulting word database can be used in studies where control for the above psycholinguistic variables is critical to the research design.

## Introduction

Words are characterized by a number of affective and lexico-semantic variables. Examples of affective variables include valence and arousal, which refer to the extent of pleasant or unpleasant feeling, and the amount of physiological response, that a word evokes respectively [1]. Valence and arousal can be rated subjectively, and their normative values are derived from rating studies [2]. On the other hand, lexico-semantic variables can be objective or subjective. An example of an objective property is word frequency, which can be assessed by how frequently a word appears in print [3,4], or more recently, in television and film subtitles [5]. Commonly studied subjective lexico-semantic variables include concreteness, imageability, and familiarity. Concreteness is the extent to which a word is tangible [6]. Imageability refers to the ease of generating a mental image for a word [6]. Familiarity refers to how frequently a word is seen, heard, or used in everyday life [7]. Like valence and arousal, values for these subjective lexico-semantic variables are obtained from rating studies and used to create population norms.

Words are commonly used as experimental stimuli in cognitive psychology studies. For example, in memory research, words are frequently used to study recollection and familiarity, processes that support recognition judgments [8,9]. While affective and lexico-semantic psycholinguistic properties of words are active areas of research on their own, they are nuisance variables that must be carefully controlled for in memory studies. This is because in memory studies, the research focus is not on the properties of the words but on memory processing. More importantly, psycholinguistic properties are known to influence memory processes. For example, research has shown that high frequency words are better recalled than low frequency words [10], while low frequency words are better recognized [11,12]. Concrete words [11], words that can easily form an image in the mind [13], and words that are high in valence or arousal [14], are also found to be better remembered. Therefore, these variables must be controlled for when words are used as experimental stimuli. This is accomplished by setting ranges for psycholinguistic variables when words are selected from databases, e.g., limiting the selection to neutral words rated between 3.5 and 4.5 on a 7-point scale, and also counterbalancing the words’ appearance across experimental conditions and/or across participants. In the methods section of research articles, it is common practice to report word length, number of syllables, and word frequency of the word stimuli selected, and how the stimuli are counterbalanced [15–18]. Although less commonly reported, affective and lexico-semantic psycholinguistic properties should be listed in the methods sections as well, since they too might influence the results, as illustrated above.

To help researchers generate suitable stimuli for experiments, it is therefore critical to have reliable and comprehensive psycholinguistic databases. Ratings for a wide range of psycholinguistic variables are available for English words and words in other European languages. Typically, ratings for affective and lexico-semantic variables are collected in separate studies, as they are often considered as different research interests. In English, a comprehensive database for affective variables is Bradley & Lang [2], which was recently vastly expanded [19]. The Bradley & Lang database has been used to build similar affective databases for other languages including Spanish [20], Italian [21], European Portuguese [22], and German [23]. Lexico-semantic databases are also available for many languages, including English [24–26], Italian [27], Spanish [28], and French [29]. Recently, there is increased interest in collecting both affective and lexico-semantic ratings within the same study, e.g., [23,30–34], because this would permit relationships between affective and lexico-semantic variables to be explored.

By comparison, there are few studies on affective or lexico-semantic characteristics of Chinese words. Although lexico-semantic ratings for single characters exist [35], to the author’s knowledge, no extensive work has been done for two-character words. Ratings for single characters are often uninformative on ratings for two-character words. This is because in Chinese, when two characters combine to form a word, the word often conveys a different meaning than either of its constituent characters. For example, the character “電” means “electricity”, while the character “腦” means “brain”. However, when the two characters combine to form the word “電腦”, it means “computer”. Therefore, ratings for two-character words cannot be simply derived from ratings of their constituent characters and have to be collected separately. There are only a few studies on psycholinguistic properties of two-character words, and no study has considered both affective and lexico-semantic variables, both objective and subjective ones, within the same study. While Ho et al. [36] and Wang et al. [37] provided affective ratings for 160 and 1500 two-character words respectively, lexico-semantic variables were not collected in these studies. More recently, Yao et al. [38] collected both affective and lexico-semantic ratings for 1100 words, but did not provide objective word frequency information for the words. As illustrated above, word frequency information is also crucial for designing psychology experiments. Although theoretically one can retrieve word frequency information from the databases from which Yao et al. [38] selected their words, namely the Chinese Affective Words System [37] and the Modern Chinese Dictionary of Commonly Used Words, these databases are not readily accessible online, thus hindering easy access to word frequency and subjective affective and lexico-semantic ratings simultaneously.

The aim of the current study was to fill this gap by establishing a database for two-character Chinese words that can be used in cognitive psychology experiments. Ratings for 292 nouns on valence, arousal, familiarity, concreteness, and imageability were collected from Cantonese speakers in Hong Kong (Cantonese is the variety of Chinese spoken in Hong Kong). These five variables were chosen because they are known to influence memory, as reviewed above. Words were selected from a word frequency database [39], which permitted word frequency information to be extracted and listed in the database created. Inter-rater reliability was calculated in order to provide a measure of quality for the ratings. Also, relationships between the various psycholinguistic variables were explored. It was expected that relationships that are well-established in other languages would be found (e.g., the strong positive correlation between concreteness and imageability [6,32,40]), which would serve as further evidence for the validity of the ratings.

## Methods

### Participants

A total of 164 young adults, recruited from the undergraduate student body of the Education University of Hong Kong via convenience sampling, participated in the study. All participants reported themselves to be native speakers of Cantonese (the Chinese dialect spoken in Hong Kong), use Traditional Chinese characters (the more widely used format of written Chinese in Hong Kong, compared to Simplified Chinese characters) for the majority of their day-to-day text communications including reading and writing, and have normal or corrected-to-normal vision. They either received extra course credit or HKD $20 for their participation. The study procedures were approved by the Human Research Ethics Committee of the Education University of Hong Kong. Written informed consent was obtained from participants before the study began.

### Materials

The word pool was generated from the word and character database of [39]. This database contains Chinese single characters and multi-character words along with frequency information, which is established from a corpus of film and television subtitles that contained 46.8 million characters and 33.5 million words. Using film and movie subtitles to assess word frequency is demonstrated to be a valid method of obtaining word frequency information [5,41], and has been used to assess word frequency for several other languages as well [42–45]. 293 two-character nouns were selected from the low- / medium-frequency section of the database, with a log frequency that ranged between 1.81 (1.91 words per million) and 2.71 (15.23 words per million). Word selection was restricted to the low to medium frequency range, because words in this frequency range were needed for a subsequent experiment reported elsewhere. Specific names (e.g., place names or person names), items that were not composed of two characters, or items that were in other parts of speech (e.g., connectives or verbs), were excluded. The final word pool had a mean log frequency of 2.29 (9.87 words per million) and standard deviation of 0.20 (1.22 words per million). Since the words in the Cai & Brysbaert [39] database were written in Simplified Chinese characters, conversion to Traditional Chinese characters was made by MS Word, and then manually checked for accuracy. The number of strokes of each character of the two-character word was counted by an online tool (https://name.longwin.com.tw/nos.php).

The 293 words were divided into five lists. The first list contained 65 words and the other four lists contained 57 words each. Each list would go on to be rated by a separate group of participants (see the Procedure section below). Five lists were set up because the word pool was large. If participants were to rate all words, long rating sessions would be needed, which would likely result in fatigue and non-compliance in task performance (e.g., making the same response for all ratings). For each list, five words were chosen at random to repeat within the list, so as to obtain a measure of internal rating consistency. Next, from the 65 unique words in the first list, eight words were chosen at random to be repeated in the other four lists, in order to establish inter-rater reliability between groups of participants. As a result, each list contained 70 words in total, unique and repeated words combined. Apart from the words that were planned to repeat within or between the five lists, one word was repeated in another list by mistake. This repeated word was removed from analyses, resulting in ratings for 292 unique words in total.

### Procedure

Each participant received one of the five lists, and rated each of the 70 words in the list along five dimensions: valence, arousal, familiarity, concreteness, and imageability. Participants rated all 70 words in one dimension first, before moving on to the next dimension, until ratings for all five dimensions were completed. This design was adopted to minimize the possibility that ratings of different dimensions would influence each other. The order of dimensions was counterbalanced between participants. Within participants, for each dimension, the order of word presentation was randomized. Participants were instructed to rate at their own pace. They were also told that there were no correct or incorrect answers, and that they should respond with their first impression. Each rating session lasted for about 25 minutes, including the time required for giving instructions.

Participants sat about 60cm away from a computer monitor and made responses using a keyboard. The study was programmed in E-prime. Words were presented in the font PMingLiU (新細明體) with a font size of 24. At the beginning of the study, participants were given a general overview of the aim of the study. Next, the definitions of the five dimensions were explained. For each dimension, two words that had received a low and a high rating in previous rating studies were provided as examples. They also served as anchors for the rating process. These example words were not part of the word list to be rated. Instructions were presented in a self-paced manner, and participants were encouraged to ask clarification questions. Before the rating of each dimension began, participants were again reminded of its definition and the two anchor examples.

### Measures

A 5-point Likert scale was used for the ratings of all dimensions. A horizontal bar with five evenly spaced response options remained on the screen throughout the rating process to remind participants about the direction of the scale. The study was performed in small groups of one to four participants, depending on the sign-up rate. Participants were instructed to press a 6^th^ key if they did not know the meaning of a word, and those words were excluded from analysis for that particular participant.

For the valence (愉悅度) dimension, instructions were adapted from [2]. Participants were asked to judge the degree to which they find the words to be pleasant, on a 5-point scale with 1 being unpleasant (不悦) and 5 being pleasant (愉快). The examples provided were the following: most people find the word “rejection” (拒絕) to be unpleasant, so they will choose 1 or 2. By contrast, most people find the word “romance” (浪漫) to be pleasant, so they will choose 4 or 5.

For the arousal (激烈度) dimension, instructions were adapted from [2]. Participants were asked to judge the degree to which they find the words to be arousing, on a 5-point scale with 1 being calm (平靜) and 5 being excited (激動). The examples provided were the following: most people find the word “leisure” (悠閒) to be calm, so they will choose 1 or 2. By contrast, most people find the word “nightmare” (惡夢) to be arousing, so they will choose 4 or 5.

For both the valence and the arousal dimensions, besides the horizontal bar that represented a 5-point scale, we adopted the rating scales of the Self-Assessment Manikin (SAM) [2,46,47] to facilitate rating. SAM is a scale made up of pictorial depictions of different levels of valence and arousal. The original 9-point scale was adapted to a 5-point scale, using the 1^st^, 3^rd^, 5^th^, 7^th^, and 9^th^ figure to represent the five levels. Increasing levels of valence were represented by a man changing from frowning to smiling, while increasing levels of arousal were represented by a man changing from sleepy to being widely awake, with an increasingly large “explosion” inside his body. Also, the language used in [2] was adopted: for the valence scale, participants were asked to choose 1 when they were “completely unhappy, annoyed, unsatisfied, melancholic, despaired, or bored” (p. 2) and to choose 5 when they were “happy, pleased, satisfied, contented, hopeful” (p. 2). If they felt “completely neutral, neither happy nor sad” (p. 2), they should choose 3. For the arousal scale, participants were asked to choose 1 when they were “completely relaxed, calm, sluggish, dull, sleepy, or unaroused” (p. 2) and to choose 5 when they were “stimulated, excited, frenzied, jittery, wide-awake, or aroused” (p. 2). If they were “not excited nor at all calm” (p. 2), they should choose 3.

For the familiarity (熟悉度) dimension, instructions were adapted from [7,26,40]. Participants were asked to judge the degree to which they find a word to be familiar, in terms of how often they encounter a word in everyday life, on a 5-point scale with 1 being unfamiliar (陌生) and 5 being familiar (熟悉). The examples provided were the following: most people encounter the word “Yucca” (絲蘭, a kind of plant) infrequently and find it less familiar, so they will choose 1 or 2. By contrast, most people encounter the word “newspaper” (報紙) frequently and find it familiar, so they will choose 4 or 5.

For the concreteness (具體性) dimension, instructions were adapted from [6]. Participants were asked to judge the degree to which they found the words to be concrete, on a 5-point scale with 1 being abstract (抽象) and 5 being concrete (具體). The examples provided were the following: most people find the word “how” (如何) to be abstract, so they will choose 1 or 2. By contrast, most people find the word “banana” (香蕉) to be concrete, so they will choose 4 or 5.

For the imageability (想象度) dimension, instructions were adapted from [6,48]. Participants were instructed to judge the ease with which they can form an image of the word in their mind, on a 5-point scale with 1 being difficult to form an image in the mind (難以在腦海裡形成影像), and 5 being easy to form an image in the mind (容易在腦海裡形成影像). The examples provided were the following: most people find it difficult to come up with a mental image for the word “taxation rate” (稅額), so they will choose 1 or 2. By contrast, most people find it easy to come up with a mental image for the word “beef” (牛肉), so they will choose 4 or 5.

### Supplementary material

The resulting ratings are listed in the Supporting Information section. In an Excel spreadsheet, the 292 words are sorted in descending order by their log frequency. English translations (performed online using Google Translate, Bing Translate, PROMT (Online-Translator.com), and LanguageWeaver (www.reverso.net)) of the words are provided for reference. The five variables are ordered from left to right in the following manner: valence, arousal, familiarity, concreteness, and imageability. For each word and for each rating dimension, the mean (M) rating for the word and its standard deviation (SD) are listed. For each word, the number of strokes of each character, as well as the word as a whole, are also listed. This property might be useful to researchers who are interested in the visual complexity of Chinese words.

Word frequency for each word is expressed in words per million (wpm), log wpm, and the Zipf value. wpm and log wpm are widely used frequency measures in psycholinguistic studies. Like log wpm, the Zipf scale is a logarithmic scale. It is derived using the formula log_10_ of the frequency per million words + 3, which can be conceptualized as log_10_ of the frequency per billion words. The Zipf value is provided alongside the widely used wpm and log wpm measures because the Zipf scale is more intuitive [44]: it typically varies between 1 to 7 like a Likert-scale, and low frequency words that have a negative value after the log_10_ frequency per million transform (low frequency words may have less than one occurrence per million words, which result in negative log values) has a more intuitive value of 1 or 2 after a log_10_ frequency per billion transform.

## Results and discussion

### Data trimming

Before the ratings were analyzed, the raw data was examined for outliers in four steps. In the first step, for each participant, the proportion of responses that had a reaction time of 300ms or below was calculated. Six participants were found to have made more than 15% of their responses in less than 300ms. They were removed from further analyses, as the high proportion of aberrantly quick responses suggested that these participants did not perform the task properly. A 300ms response is considered as aberrantly quick, as per standard practice in previous studies [49,50]. Also, as in these two previous studies, we did not set an upper limit for reaction time because speed was not emphasized in the instructions.

In the second step, participants who reported an aberrantly large number of words that they did not know were excluded from further analysis. Considering that the majority (83%) of participants did not have any such responses, those who reported that they did not know more than 10 words out of a total of 70 were considered as aberrant. Eight participants were excluded by this criterion. Three additional participants were excluded because they had more than 10 such responses for all rating dimensions combined. Although within a single dimension, the number of words that they reported that they did not know was small, those words were not consistent across dimensions, casting doubts on these participants’ compliance with task instructions.

After the first two steps, 147 participants remained. Of these participants, only 17 reported a word / words that they did not know. In these 17 participants, they reported an average of 0.82 ± 0.58 words (mean ± S.D.) per rating dimension that they did not know. This number is considered reasonable. Next, for these remaining participants, trials under 300ms were excluded from analysis. This led to a removal of 523 ratings out of a total of 51,450 ratings across all dimensions across all participants.

In the third step, the mean and standard deviation of the ratings were calculated for each word. Responses that were outside the range of ± 2.5 S.D. were removed from further analysis, resulting in a further removal of 585 ratings across all dimensions across all participants.

In the last step, internal reliability was calculated for each subject, using the five words that were rated twice within participants. As in [48], participants with consistency that was lower than 0.2, as indicated by the Pearson correlation coefficient, were removed from further analysis. Four participants were removed by this criterion. The mean and standard deviation of consistency in the remaining 143 participants was 0.74 ± 0.18, demonstrating good consistency.

Therefore, the final dataset consisted of ratings from 143 participants (87% of participants tested). Each word received at least 22 and at most 33 valid ratings. For valence, arousal, familiarity, concreteness, and imageability, each word received 27.8 ± 3.10, 28.3 ± 2.99, 27.3 ± 2.74, 28.2 ± 2.97, and 28.4 ± 3.02 (mean ± S.D.) valid ratings respectively.

### Reliability

First, rating consistency within each sample of participants (25 or more participants per sample, who rated the same word list) was assessed. Adopting the same approach as [32], the inter-rater reliability of the ratings was assessed by calculating the intra-class correlation coefficients (ICCs). The ICCs were calculated for the five dimensions separately. For each dimension, an ICC was obtained for each sample of participants. Then, a mean ICC for each dimension was obtained by averaging the ICCs of the five samples of participants. The mean and standard deviation of the ICCs for each dimension are: valence: 0.95 ± 0.02, arousal: 0.86 ± 0.04; familiarity: 0.77 ± 0.08; concreteness: 0.84 ± 0.04; and imageability: 0.81 ± 0.07. Overall, the ICCs were high for all five dimensions, demonstrating high inter-rater reliability in our samples. In order to provide a standardized measure of variability, the coefficient of variation (CV) was also computed for each rating dimension. The CVs were 3%, 5%, 10%, 5%, and 8% for valence, arousal, familiarity, concreteness, and imageability respectively. Therefore, both the unstandardized and standardized measures of variability indicate that valence was the most consistently rated dimension, while familiarity was the least consistently rated, although still reaching a satisfactory level. The finding that valence has a more consistent rating than arousal is consistent with previous studies for many languages [20,22,32,51,52].

Next, rating consistency among the five different samples of participants was assessed by calculating the rating consistency of the eight words that repeated across the different samples of participants. An ICC was calculated for each dimension. The ICCs were 0.99, 0.98, 0.82, 0.98, and 0.98 for valence, arousal, familiarity, concreteness, and imageability respectively, demonstrating highly consistent ratings for the five samples of participants.

### Relationship between variables

Pearson correlations were calculated for all possible pair-wise combinations of variables. There are several notable relationships (Table 1). First, concreteness had a strong positive correlation with imageability (r = .88, p < 0.001). The same strong positive correlation has been found in English [6,40], Spanish [32], French [30], European Portuguese [53], and very recently, Chinese [38]. The convergence between several languages indicates that, across these different languages, in general it is easier to form a mental image for concrete words than for abstract words. This finding is consistent with the dual-coding theory [54,55], which postulates that two systems, one verbal-based and the other imagery-based, are involved in the representation of the semantics of a stimulus. According to this theory, concrete words are processed faster than abstract words, a phenomenon known as the concreteness effect [55], because while abstract words are coded by the verbal-based system only, concrete words can be coded by both the verbal- and the imagery-based systems. The additional imagery-based coding provides an additional form of representation to facilitate processing.

**Table 1:** Correlation between variables.

	Valence	Arousal	Familiarity	Concreteness	Imageability	Word frequency (log wpm)	Number of strokes
Valence	—	-.20**	.38***	-.12*	-.01	.03	-.00
Arousal		—	-.11	-.02	.02	-.01	.10
Familiarity			—	.34***	.41***	.16**	.07
Concreteness				—	.88***	.21***	.02
Imageability					—	.09	-.00
Word frequency (log wpm)						—	-.13*
Number of strokes							—

* p < 0.05

** p < 0.01

*** p < 0.001

Pearson correlation coefficients for all pair-wise combinations of valence, arousal, familiarity, concreteness, imageability, word frequency (measured in log words per million (wpm)), and number of strokes (of both characters of the word summed together).

It is important to highlight that, although concreteness and imageability are highly correlated, they are not identical constructs, as several authors advocated in recent studies [53,56–58]. Guasch [32] found that while words tend to co-vary in these two dimensions, there are also words that are high in concreteness but low in imageability (e.g., tuberculosis), and words that are low in concreteness but high in imageability (e.g., issue). Here in this study, it was found that concreteness and imageability each had overlapping but different patterns of correlations with other affective and lexico-semantic variables. Specifically, although both concreteness and imageability correlate with familiarity, only concreteness is found to further correlate with valence and word frequency, further revealing that concreteness and imageability are overlapping but not synonymous constructs. Their overlap is likely because one of the decision criteria for concreteness is whether a word describes an object that exists in real life, which naturally corresponds to whether the word can arouse visual imagery. This criterion therefore overlaps with the decision criterion for imageability, and explains their shared variance. On the other hand, their non-overlapping variances invite further research into what contribute to the decision criteria when people make these rating judgments. For example, Connell & Lynott [56] proposed whether a word represents a typical exemplar of a concrete category (objects, materials, etc.) also contributes to its concreteness rating.

Both concreteness and imageability correlate positively with familiarity. The same correlations were found in previous studies: for the moderate positive correlation between concreteness and familiarity (r = .34, p < 0.001), a similar correlation was found in [38]; for the moderate positive correlation between imageability and familiarity (r = .41, p < 0.001), similar-sized correlations have been obtained in [26,31,32,38]. The implication of these correlations is that more concrete and more imageable words tend to be perceived as more familiar.

Besides subjective familiarity, concreteness also had a moderate positive correlation with objective word frequency (r = .21, p < 0.001). Previous studies have also identified a positive correlation between concreteness and printed frequency [6,59]. Together, they illustrate that more concrete words not only are perceived to be more familiar, but they also objectively occur more frequently in the language.

Concreteness was found to correlate with valence negatively (r = -.12, p < 0.05). This effect was also observed in [60], and the size of the correlation is highly similar (r = -.11 in [60]). It means that words that are subjectively perceived to be more pleasant are also perceived to be more abstract. This finding highlights the importance of taking concreteness into consideration when investigating the processing of emotional words, and vice versa. Indeed, it has been shown that concreteness influences the processing of emotional words [58,61,62]. Broadly speaking, this finding demonstrated that affective variables correlate with lexical variables, and highlights the importance of investigating the relationship between the two in future studies.

Familiarity correlated positively with valence (r = .38), consistent with [31], where they further demonstrated that the correlation was driven by a response bias, in that participants were more willing to say that they were familiar with positive words. Familiarity also correlated positively with word frequency (r = .16), consistent with several previous studies [24,26,31], although the size of the correlation is smaller than in these studies, which had r’s of .35 or above. The small correlation is likely due to the restricted frequency range of the words included in this study, making it difficult to find a large correlation. In addition, subjective familiarity for low frequency words is likely influenced by word prevalence, a measure of the number of people in the population who know the word [63].

**Fig 1:**
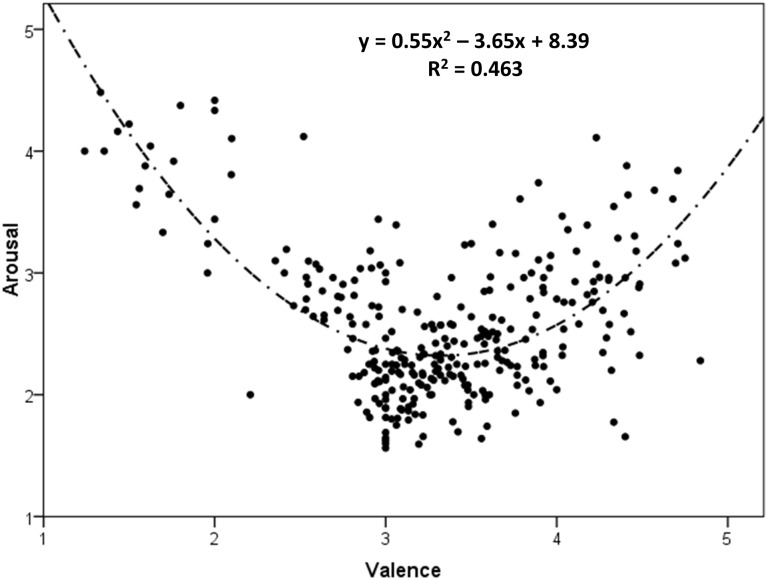
Distribution of the mean ratings (provided by at least 22 participants) for the 292 words in the valence and arousal dimensions.

Lastly, we examined whether there is a quadratic relationship between valence and arousal, as has been widely reported. Bradley & Lang [2] observed a quadratic relationship between valence and arousal, where highly positive and negative words had higher ratings on arousal. Since then, the same effect has been observed many times in rating studies in different languages, including Finnish [51], Spanish [20,32,64,65], German [34,66], European Portuguese [22], and Chinese [36]. A step-wise regression analysis with a linear and a quadratic term was performed, with valence and its square as the independent variables and arousal as the dependent variable. Although a linear model was significant (R = 0.20, F(1,290) = 11.61, p < 0.005), it only accounted for 3.8% of the variance. By contrast, the quadratic model explained an additional 42.4% of the variance (R = 0.68, F(2,289) = 124.20, p < 0.001), hence demonstrating a better fit for the quadratic model. Fig 1 shows this typical U-shaped function between valence and arousal.

It is of particular interest to compare the current study with Yao et al. [38], since both studies acquired ratings on valence, arousal, familiarity, concreteness, and imageability for Chinese words, and only differed in two aspects: Yao et al. [38] did not provide word frequency information, while the current study did not collect context availability ratings. Words in the two studies only overlapped to a small extent: among the 292 words rated in the current study, only 26 were rated by Yao et al. Despite the small overlap, the highly statistically significant relationships between variables found in the current study (p’s < 0.01) were all found in Yao et al. [38], including the quadratic relationship between valence and arousal, and the correlations between concreteness and familiarity, between concreteness and imageability, between familiarity and imageability, and between familiarity and valence, indicating that these relationships are likely stable for Chinese words in general.

### Potential uses of the current database

As mentioned in the Introduction, the motivation for conducting this study was to create a database that can be used to generate sets of Chinese words that are matched on various psycholinguistic variables. To show that the database can be used for this purpose, analyses were conducted in which random samples of words were drawn from the database, split into two halves, and tested to see if the two halves were indeed matched on the various psycholinguistic variables. This analysis approach mimicked the typical way the database would be used to generate stimuli for experiments. Seven separate analyses were conducted, using varying sample sizes of 120, 140, 160, 180, 200, 220, and 240 words. These sample sizes were chosen because they are representative of the number of stimuli typically required for small-scale memory experiments [e.g., Experiment 1 in Hennessee et al. [67] presented 180 words in total; Hoppstädter et al. [68,69] presented 200 words and 100 words in their studies respectively; Ozubko et al. [70] presented 120 words in total]. Each sample was split into two sets and compared against each other (instead of splitting into three or more sets) because admittedly, the database has only 292 words and is unlikely to have enough stimuli for more complex experimental designs. The small number of words in the database is currently its major limitation. Now, a sample size of 120 words will be used as an example to illustrate how the analyses were conducted. First, 120 words were randomly selected from the database and divided into two sets of 60 words each. Then, seven t-tests, one for each psycholinguistic variable (valence, arousal, familiarity, concreteness, imageability, word frequency, and number of strokes), were conducted to compare the two sets on these dimensions. If none of the p values was below .05, it would mean that there was not enough evidence to suggest that the two sets were different from each other on any of the dimensions, and it would be deemed appropriate to present the two sets as matched stimuli in two experimental conditions. This process of selecting a random sample and then performing a t-test on each psycholinguistic dimension was repeated for 10000 times to see how often a random sample drawn from the database would produce two halves that did not statistically differ on any of the seven dimensions. For a sample size of 120 words, out of 10000 samples, when each dimension was considered alone, roughly 90% of the samples produced two halves that were matched on that particular dimension (percentage of matching halves for valence: 90.1%; arousal: 89.6%; familiarity: 90.5%; concreteness: 90.1%; imageability: 90.0%; word frequency: 89.7%; number of strokes: 90.2%). Critically, when all seven variables are considered together, 52.2% of the 10000 samples were matched on all of the variables (i.e., did not have a significant p-value in any of the seven t-tests). Similar results were obtained across all sample sizes: the percentage of samples that were matched on all seven variables ranged between 51.4% and 52.5% for the seven sample sizes tested. These results suggest that, when a random sample of words is drawn from the database and split into two halves, it can be expected that the two halves will be matched on all the variables slightly more than half of the time. Considering that as many as seven variables are matched, the success rate is quite high and that the database can indeed be used to create matched stimuli sets easily.

Apart from creating stimuli sets that are matched on several psycholinguistic variables, some researchers might be interested in manipulating one or more psycholinguistic variables while controlling for the others. For example, in studies where the aim was to investigate how lexical decision times were affected by word concreteness [58], researchers had to manipulate concreteness while holding other variables like imageability and familiarity constant, and therefore required words that differ in concreteness but not other dimensions. To see whether researchers can use the database for this purpose, a separate analysis was done for each psycholinguistic dimension to see if that variable could be manipulated while controlling for the other variables. Now, analysis for the valence dimension will be used to illustrate how the analyses were done in general. First, words were rank-ordered according to valence. Next, two stimuli sets, each with a set size of n words, were formed by the first and last n words in the ranked-ordered word list. These stimuli sets corresponded to sets of high and low valence words respectively. The two sets were then compared to see 1) whether they were statistically different from each other in the valence dimension (where the ideal outcome would be that the two sets were statistically different, meaning that valence was successfully manipulated), and 2) whether they were statistically different in the other six dimensions (where the ideal outcome would be that they were not statistically different, meaning that they were matched on these dimensions). These statistical comparisons were conducted via t-tests. This analysis approach mimicked how researchers would extract words with extreme values in one dimension in order to manipulate that dimension, and then checking to see if the other variables are matched. The analysis was performed for stimuli set sizes (n) that varied between two words and 146 words. An n of 146 words was the maximum number of words that the high and low sets could have, since the database has 292 words total (an n of 146 words is equivalent to a median split). A small n is admittedly trivial, as it is unlikely that such a small number of stimuli would be sufficient for any experiment. Nonetheless, the analyses were done for completeness. For all of the psycholinguistic variables, it was found that as set size varied, although the first criterion was always met, that is, the high and low sets always differed in the dimension in which the words were manipulated, the second criterion was rarely met—the percentage of set sizes that were matched on all other variables when valence was manipulated was 0.7%, and they were 0%, 0%, 0%, 6.2%, and 11.0% respectively when familiarity, concreteness, imageability, word frequency, or number of strokes was manipulated. The only exception was arousal: when it was manipulated, 57.9% of the set sizes were also controlled for the other six variables (e.g., when the high and low arousal word set contained 81 to 146 words each; see Supporting Information S2 for a complete listing of set sizes that met both criteria). In summary, these analyses showed that researchers who are interested in manipulating arousal would find this database useful, when their study design requires high and low arousal words of certain set sizes. However, for researchers who would like to manipulate valence, familiarity, concreteness, imageability, word frequency, or number of strokes, it is unlikely that they can use the database to generate stimuli sets that differ in these dimensions while controlling for the others. This is a limitation of the current database, mostly due to the small number of words it has. A much larger database will be needed in order to manipulate certain variables while controlling for the others. This is especially true for variables that are correlated (e.g., concreteness and imageability). For these variables, the correlation makes it more difficult to find words that differ in one dimension but not the other. Increasing the size of the database can ameliorate this problem by increasing the chance that non-typical words (e.g., words high in concreteness but low in imageability) are included. Also, having more words in the database can increase the number of words in the extreme ends so that researchers can maximize the difference along a certain dimension in their manipulation. Finally, the current database only includes seven variables and the words are within a restricted range of word frequency; expanding the database to include other variables like age of acquisition, as well as covering a wider range of word frequency would make the database useful for more research purposes.

## Conclusion

In conclusion, ratings of valence, arousal, familiarity, concreteness, and imageability for 292 two-character Chinese nouns were obtained from a Cantonese-speaking population, and listed in the resulting database. Word frequency and number of strokes were also listed. The ratings obtained showed good reliability. Several notable relationships among the variables were found, all of which have been found in similar rating studies for other languages, further demonstrating the validity of the current ratings. The present database will be useful for researchers who would like to control for psycholinguistic properties in their studies, when their studies require relatively few stimuli with few experimental conditions. For researchers who would like to manipulate certain psycholinguistic variables while controlling for others, the current database has limited utility, and can only be used to manipulate arousal. Future studies should expand the database to offer a wider word choice, so that studies with more complex designs and require more stimuli can also use the database to control for or manipulate one or more psycholinguistic variables.

## Supporting information

S1 File Word ratings. In an Excel spreadsheet, the 292 words are sorted in descending order by their log frequency. English translations (performed online using Google Translate, Bing Translate, PROMT (Online-Translator.com), and LanguageWeaver (www.reverso.net)) of the words are provided for reference. The five variables are ordered from left to right in the following manner: valence, arousal, familiarity, concreteness, and imageability. For each word and for each rating dimension, the mean (M) rating for the word and its standard deviation (SD) are listed. For each word, the number of strokes of each character, as well as the word as a whole, are also listed. Word frequency for each word is expressed in words per million (wpm), log wpm, and the Zipf value, the definition of which can be found in the Supplementary material section of the main text.
(XLSX)

S2 File Results of analyses on whether a psycholinguistic variable could be manipulated while controlling for other variables are presented in a table listing stimuli set sizes (rows) by psycholinguistic variables (columns). Set sizes for which a certain psycholinguistic variable was successfully manipulated while controlling for the other six variables are marked with “1”. For example, for the “1” in the cell where valence and a set size of two intersect, it means that the database of 292 words was first rank-ordered by valence, and then two stimuli sets of two words each were formed by the first and last two words in the rank-ordered list. T-tests comparing the two sets on all dimensions indicated that they are statistically different in valence, while matched on all the other six variables.
(XLSX)
